# Multiparameter MRI Model With DCE-MRI, DWI, and Synthetic MRI Improves the Diagnostic Performance of BI-RADS 4 Lesions

**DOI:** 10.3389/fonc.2021.699127

**Published:** 2021-10-15

**Authors:** Shi Yun Sun, Yingying Ding, Zhuolin Li, Lisha Nie, Chengde Liao, Yifan Liu, Jia Zhang, Dongxue Zhang

**Affiliations:** ^1^ Department of Radiology, Yunnan Cancer Center, The Third Affiliated Hospital of Kunming Medical University, Yunnan Cancer Hospital, Kunming, China; ^2^ Magnetic Resonance Imaging Research, General Electric Healthcare (China), Beijing, China; ^3^ Department of Radiology, Third People's Hospital of Yunnan Province, Kunming, China

**Keywords:** breast cancer, synthetic magnetic resonance imaging (syMRI), mapping, relaxation time, quantitative imaging

## Abstract

**Objectives:**

To evaluate the value of synthetic magnetic resonance imaging (syMRI), diffusion-weighted imaging (DWI), DCE-MRI, and clinical features in breast imaging–reporting and data system (BI-RADS) 4 lesions, and develop an efficient method to help patients avoid unnecessary biopsy.

**Methods:**

A total of 75 patients with breast diseases classified as BI-RADS 4 (45 with malignant lesions and 30 with benign lesions) were prospectively enrolled in this study. T1-weighted imaging (T1WI), T2WI, DWI, and syMRI were performed at 3.0 T. Relaxation time (T1 and T2), apparent diffusion coefficient (ADC), conventional MRI features, and clinical features were assessed. “T” represents the relaxation time value of the region of interest pre-contrast scanning, and “T+” represents the value post-contrast scanning. The rate of change in the T value between pre- and post-contrast scanning was represented by ΔT%.

**Results:**

ΔT1%, T2, ADC, age, body mass index (BMI), menopause, irregular margins, and heterogeneous internal enhancement pattern were significantly associated with a breast cancer diagnosis in the multivariable logistic regression analysis. Based on the above parameters, four models were established: model 1 (BI-RADS model, including all conventional MRI features recommended by BI-RADS lexicon), model 2 (relaxation time model, including ΔT1% and T2), model 3 [multi-parameter (mp)MRI model, including ΔT1%, T2, ADC, margin, and internal enhancement pattern], and model 4 (combined image and clinical model, including ΔT1%, T2, ADC, margin, internal enhancement pattern, age, BMI, and menopausal state). Among these, model 4 has the best diagnostic performance, followed by models 3, 2, and 1.

**Conclusions:**

The mpMRI model with DCE-MRI, DWI, and syMRI is a robust tool for evaluating the malignancies in BI-RADS 4 lesions. The clinical features could further improve the diagnostic performance of the model.

## Introduction

The breast imaging–reporting and data system magnetic resonance imaging (BI-RADS-MRI) lexicon proposed by the American College of Radiology (ACR) is a reference for MRI interpretation of breast lesions. It provides MRI descriptors, such as signal intensity, morphology, and enhancement kinetics, which are recommended in combination to evaluate the final BI-RADS classification (1). The lesions that do not have typical malignant signs but have sufficient suspicious manifestations are classified as BI-RADS 4 (>2% but <95% likelihood of malignancy). Such a wide range prompts the patients to undergo an unnecessary histological biopsy.

Several studies have confirmed that dynamic contrast-enhanced magnetic resonance imaging (DCE-MRI) is an effective “problem-solving” tool for further evaluation of BI-RADS category 4 findings that are classified by mammography or ultrasonography (US) (2–5). When mammography or/and US remains inconclusive yet suspicious, additional imaging with MRI is the next recommended plan. However, most descriptors recommended by BI-RADS are based on the subjective qualitative evaluation depending on the experience and level of different observers. In particular, the judgment of signal strength is affected by the subjective difference and image contrast. On the other hand, due to an overlap of morphological and kinetic enhancement features between benign and malignant lesions, the specificity is limited (6, 7). When MRI becomes the last step of non-invasive imaging examination, patients still judged as BI-RADS 4 can only choose biopsy for diagnosis (8).

Diffusion-weighted imaging (DWI), magnetic resonance spectroscopy (MRS), and other quantitative imaging techniques in diagnosing breast BI-RADS 3–5 lesions have been explored extensively to aid in diagnosis specificity. Relaxation time could be another quantitative MR method for the diagnosis of breast diseases (9–15). Previous studies have shown that quantitative longitudinal relaxation time (T1) and transverse relaxation time (T2) values could assess the histopathology of breast diseases and, thus, be used as a potential biomarker of biopsy (16, 17). Some studies have explored the use of quantitative relaxation time in breast disease diagnosis, histopathological grading, neoadjuvant chemotherapy evaluation, and Ki-67 status prediction (18–23). However, only a few studies have focused on the relaxation time in breast BI-RADS 4 lesions, and the diagnostic value of relaxation time in this specific population needs further investigation.

Recently, a promising quantitative imaging technique, synthetic MRI (syMRI), has been proposed. It is based on the quantification of relaxation times by multi-echo acquisition of a saturation-recovery using the turbo spin-echo readout (QRAPMASTER) method. In addition, a multi-delay multi-echo (MDME) approach, magnetic resonance image compilation (MAGiC), and a slice shift between the saturation pulse and acquisition facilitate efficient quantification of T1 and T2 by syMRI in a single scan, as well as the subsequent synthesis of multiple contrast-weighted images. The quality of these synthetic images is sufficient for diagnosis (24), and the acquisition time of syMRI is only one-third of the traditional quantitative technology (18) as it applies the multi-echo spin-echo (MESE) method to quantify the relaxation time. A previous study pointed out that syMRI is useful for the evaluation of breast cancer by simultaneous acquisition of several quantitative physical properties. Moreover, the relaxation time obtained by syMRI has shown an excellent correlation with that by traditional mapping (18). Herein, we analyzed the contribution of conventional MR features recommended by BI-RADS, quantitative relaxation time provided by syMRI, apparent diffusion coefficient (ADC) obtained by DWI, and clinical features in the diagnosis of breast BI-RADS type 4 lesions. Additionally, we aim to establish a multi-parameter (mp) combination model and present it with a visual nomogram and develop an efficient method for diagnosing BI-RADS 4 breast lesions to avoid unnecessary biopsies in patients.

## Materials and Methods

### Study Population

The present study was conducted on 255 female patients who underwent breast MRI in our institution at the Yunnan Cancer Hospital from July 2019 to September 2019. The study was approved by the institutional ethics board of the hospital (No. KY2019102), and informed consent was obtained from all individual participants. The inclusion criteria were as follows: (1) no previous operation or any treatment; (2) before and after contrast injection, all patients underwent complete syMRI and routine MRI examination; and (3) BI-RADS final assessment as category 4 at MRI. The exclusion criteria were as follows: (1) patients with incomplete clinical or pathological data; (2) patients with insufficient MRI image quality for quantitative measurement; (3) non-mass lesions; and (4) patients with both benign and malignant lesions.

### MRI Acquisition

All MR examinations were performed on a 3.0-T MRI system (Signa Pioneer, GE Healthcare, Milwaukee, WI, USA) using a 16-channel phased-array breast surface coil, with the patient in the prone position. The imaging protocols are described in **Appendix 1**. Firstly, before contrast injection, all patients underwent conventional MRI (including T1WI, T2WI, and DWI), followed by syMRI (MAGiC). Secondly, 20 ml of Gd-DTPA-BMA (Omni-Scan, GE Healthcare, Ireland) was injected at a rate of 2.0 ml/s and then flushed with 20 ml of saline. Next, DCE-MRI was performed, followed by syMRI. The scanning parameters of syMRI pre- and post-contrast injection were identical. The scan parameters of all sequences are listed in **Table 1**.

**Table 1:** Scan parameters for all sequences.

Parameters	T1WI	T2WI	DWI	DCE-MRI	syMRI
Sequence	FSE	STIR	Single shot echo	Spoiled gradient echo	MAGiC
Orientation	Ax	Ax	Ax	Ax	Ax
Fat suppression	No	Yes	Yes	Yes	No
TR (ms)	8.6	5,600	4,800	4.43	4,000
TE (ms)	4.7	56	81	1.5	19.2/86.2
Slice thickness (mm)	5	5	5	1.2	5
No. of sections	34	34	34	144	34
FOV (cm)	36×36	36×36	36×18	36×36	36×36
Matrix	320×224	256×256	220×110	256×384	260×260
Bandwidth (Hz/pixel)	390.6	651.0	250.0	1,116.1	213.7
Acceleration factor	2	2	2	2	2
b-value (s/mm ^2^)	-	-	0/800	-	-
TA (min)	1.62	3.17	2.5	5.5 (5 phases)	5.1

FSE, fast spin echo; STIR, short TI inversion recovery; FOV, field of vision; TA, time of acquisition. "-", means not applicable.

### Image Analysis

#### Quantitative MRI Characteristics

Two radiologists (A and B, with 10 and 8 years of experience in breast imaging, respectively), blinded to the pathology results, clinical data, and other imaging findings, reviewed all the images. The syMRI data were further processed using syMRI 8.0 software (SyMR, Linköping, Sweden) to generate T1 and T2 relaxation maps. All the images have been processed on the Advantage Windows workstation (GE Healthcare, AW 4.4) as follows: (1) The relaxation maps and conventional MRI weighted images were observed to determine the location of the lesion, and a region of interest (ROI) was drawn manually on one of the relaxation maps along the margin of the tumor on a single section image with maximum diameter to cover the largest area of the lesion. (2) Then, the ROI was mapped to the other relaxation maps through the automatic alignment of MAGiC software to ensure the consistency of the ROI range. T1 and T2 relaxation maps were obtained simultaneously in one scan by syMRI, and the scanning parameters of syMRI were altered pre- and post-contrast injection. (3) The sections of DWI and syMRI scanning were identical, and hence, the number of ADC and relaxation maps was also similar. The ROI was drawn on the ADC map by manual alignment, the delineated area was consistent with the area on the relaxation map, and the area error was controlled within 0.1 cm ^2^. (4) The values of quantitative parameters (T1, T2, and ADC) of the ROIs were calculated automatically *via* MAGiC and DWI post-processing software, and the median values were utilized for statistical analysis. “T” represents the relaxation time value of the ROI pre-contrast scanning, and the “T+” represents the value post-contrast scanning. ΔT% (ΔT%=[(“T”–”T+”)/”T”]×100) represented the relative change rate in T value between pre- and post-contrast scanning. The area of ROI was 1–6.5 cm ^2^ according to the size of the tumor. The largest lesion would be involved in this study when more than one lesion was detected in the breasts. After 2 weeks, both observers, blinded to their previous results, independently remeasured the data similarly.

#### Conventional MRI Characteristics

According to the 5th edition BI-RADS MRI lexicon (1), the two observers independently evaluated the conventional MRI characteristics including size (longest diameter), shape (round or oval and irregular), margin (circumscribed, irregular, and spiculated), internal enhancement pattern (homogeneous, heterogeneous, rim enhancement, and dark internal septations), the initial phase of time-intensity curves (TIC) (slow, medium, and fast), delayed phase of TIC [persistent (I), plateau (II), and washout (III)], breast composition defined by the amount of fibroglandular tissue (FGT) (fat, scattered, heterogeneous, or extreme), background parenchymal enhancement (BPE) (minimal, mild, moderate, and marked), peritumoral edema (yes or no), and associated features (lymph nodes enlargement, nipple retraction and invasion, skin retraction and invasion, pretoralis muscle invasion, and architectural distortion). In the case of discrepancy in the opinions of the two observers, the judgment of the third doctor with higher qualifications prevails.

#### Clinical Characteristics

Clinical data, such as age, menarche age, body mass index (BMI), menopausal state, fertile state, and family history (first-degree or second-degree relatives with breast cancer), were obtained from patients’ medical records. Other characteristics were obtained by reviewing patients’ biochemical reports, including the status of carcinoembryonic antigen (CEA), carbohydrate antigen 125 (CA125), CA153, and ferritin (positive or negative).

### Statistical Analysis

All statistical analyses were carried out using the statistical packages R (The R Foundation; http://www.r-project.org; version 3.4.3) and Empower (R) (www.empowerstats.com, X&Y solutions, Inc. Boston, MA, USA). Firstly, the Kolmogorov–Smirnov test was applied to assess the normality of continuous data. Categorical data were presented as frequencies and percentages (*N* and %, respectively). Continuous data were summarized as means ± standard deviation (M ± SD) or median and interquartile ranges M (Q1, Q3), depending on the distribution.

### Construction of the Prediction Models

The variables between benign and malignant groups were compared using the Mann-Whitney *U* test, Student’s *t*-test, chi-square test, and Fisher’s exact test. The variables with *p* < 0.05 in univariate analysis were entered into the multivariable logistic regression analysis. Based on the minimal Akaike’s information criterion (AIC), the multivariable logistic regression analysis was used to screen the independent variables associated with malignancy. Thus, various predictive models were constructed based on these independent variables.

### Evaluation of Model Effectiveness

The discriminative ability of variables was examined using the area under the receiver operating characteristics (ROC) curve (AUC). The maximum of Youden’s index = (sensitivity + specificity) −1 was used as a critical point to identify benign and malignant breast lesions. The net reclassification index (NRI) was used to evaluate the diagnostic efficiency among the models. The decision curve analysis (DCA) evaluated the clinical utility of the model, while the calibration curve assessed the fitness of the model. Limited by the small sample size, the bootstrap resampling method (1,000 times) was used for internal verification of the model.

The interobserver and intraobserver consistencies for all quantitative parameters between the two radiologists were evaluated with the intraclass correlation coefficient (ICC) and the Bland–Altman analysis.

## Results

According to the above inclusion and exclusion criteria, a total of 75 patients (45 with malignant lesions and 30 with benign lesions) were included in this study (**Appendix 2**). All the lesions were confirmed by biopsy or pathology after surgery (**Appendix 3**).

### Imaging and Clinical Characteristics to Distinguish Breast Cancer From Benign Diseases in BI-RADS 4 Lesions

Univariate analysis showed significant differences in T1, ΔT1%, T2, T2+, ΔT2%, ADC, age, BMI, menopausal state, CA153, margin, FGT, and internal enhancement pattern between benign and malignant groups (**Table 2**). ΔT1%, T2, ADC, age, BMI, menopause, irregular margins, and heterogeneous internal enhancement pattern were identified as independent variables for breast cancer diagnosis in the multivariable logistic regression analysis (**Table 3**).

**Table 2:** Comparison of clinical and imaging characteristics between benign and malignant groups.

Variables	Benign N = 30	Malignant N = 45	p-value	Variables	Benign N = 30	Malignant N = 45	p-value
T1	1,240.80 (1,111.19,1,366.09)	1,376.55 (1,299.57,1,544.15)	0.002*	Size	2.30 (1.42,3.38)	2.50 (2.00,3.70)	0.396
T1+	313.50 (303.60,336.12)	317.06 (310.69,320.82)	0.829	Age	44.00 (31.00,55.00)	49.22 (45.00,54.00)	0.048*
ΔT1%	74.20 ± 3.38	77.18 ± 2.59	<0.001*	BMI	22.34 ± 2.76	23.78 ± 2.49	0.021*
T2	92.57 (90.19,96.14)	84.63 (81.34,86.50)	<0.001*	Menarche Age	13.50 (12.00,14.00)	13.00 (12.00,14.00)	0.563
T2+	88.66 (84.94,90.85)	79.73 (76.21,81.80)	<0.001*	Menopausal State			0.017*
ΔT2%	5.39 (4.45,6.08)	5.94 (5.43,6.42)	0.022*	Pre	12 (40.00%)	7 (15.56%)	
ADC	1.25 ± 0.09	1.10 ± 0.15	<0.001*	Post	18 (60.00%)	38 (84.44%)	
Shape			0.559	Fertile State			0.142
Round or oval	10 (33.33%)	18 (40.00%)		No	3 (10.00%)	1 (2.22%)	
Irregular	20 (66.67%)	27 (60.00%)		Yes	27 (90.00%)	44 (97.78%)	
Margin			0.023*	Family History			0.093
Circumscribed	15 (50.00%)	11 (24.44%)		Yes	1 (3.33%)	7 (15.56%)	
Irregular	15 (50.00%)	34 (75.56%)		No	29 (96.67%)	38 (84.44%)	
Spiculated	0 (0.00%)	0 (0.00%)		CEA			0.790
Internal Enhancement Pattern			0.013*	Negative	26 (86.67%)	38 (84.44%)	
Homogeneous	5 (16.67%)	3 (6.67%)		Positive	4 (13.33%)	7 (15.56%)	
Heterogeneous	14 (46.67%)	28 (62.22%)		CA153			0.059
Rim enhancement	6 (20.00%)	14 (31.11%)		Negative	30 (100.00%)	40 (88.89%)	
Dark internal septations	5 (16.67%)	0 (0.00%)		Positive	0 (0.00%)	5 (11.11%)	
Initial Phase of TIC			0.041*	CA125			0.810
Slow	5 (16.67%)	6 (13.33%)		Negative	29 (96.67%)	43 (95.56%)	
Medium	20 (66.67%)	19 (42.22%)		Positive	1 (3.12%)	2 (4.44%)	
Fast	5 (16.67%)	20 (44.44%)		Peritumoral Edema			0.093
Delayed Phase of TIC			0.434	No	30 (100.00%)	41 (91.11%)	
Persistent (I)	4 (13.33%)	3 (6.67%)		Yes	0 (0.00%)	4 (8.89%)	
Plateau (II)	23 (76.67%)	34 (75.56%)		Associated Features			1.000
Washout (III)	3 (10.00%)	8 (17.78%)		No	28 (93.33%)	42 (93.33%)	
FGT			0.928	Yes	2 (6.67%)	3 (6.67%)	
Fat	3 (10.00%)	6 (13.33%)		BPE			0.742
Scattered fibroglandular tissue	11 (36.67%)	16 (35.56%)		Minimal	3 (10.00%)	4 (8.89%)	
Heterogeneous fibroglandular tissue	9 (30.00%)	11 (24.44%)		Mild	19 (63.33%)	24 (53.33%)	
Extreme fibroglandular tissue	7 (23.33%)	12 (26.67%)		Moderate	6 (20.00%)	11 (24.44%)	
				Marked	2 (6.67%)	6 (13.33%)	

BMI, body mass index; TIC, time–signal intensity curve; CEA, Carcinoembryonic antigen; ADC, apparent diffusion coefficient.

Categoric data were calculated using chi-square test or Fisher’s exact test. Continuous data were calculated using Mann–Whitney U test or Student’s t-test, and a significant difference of p < 0.05 was used. *P < 0.05.

**Table 3:** Parameters associated with breast cancer diagnosis in multivariable logistic regression analysis.

Variables	Multivariable logistic regression analysis		Variables	Multivariable logistic regression analysis	
	OR (95% CI)	p-value		OR (95% CI)	p-value
T1	0.0006 (0.0000, inf.)	0.5819	Margin		
ΔT1%	1.9469 (1.2334,3.0732)	0.0042*	Circumscribed	0.2167 (0.0593,0.7927)	0.0208*
T2	0.7766 (0.6597,0.9142)	0.0024*	Irregular	Ref.	1.0
T2+	NA	NA	Spiculated	–	–
ΔT2%	1.8690 (0.9008,3.8777)	0.0930	Internal Enhancement Pattern		
ADC	0.0001 (0.0001,0.0009)	0.0012*	Homogeneous	0.1613 (0.0262,0.9915)	0.0489*
Age	1.0618 (1.0072,1.1193)	0.0257*	Heterogeneous	Ref.	1.0
BMI	1.2956 (1.0471,1.6030)	0.0171*	Rim enhancement	1.1719 (0.2643,5.1959)	0.88346
Menopausal State			Dark Internal Septations	0.0000 (0.0000, inf.)	0.9945
Pre	0.2529 (0.0721,0.8868)	0.0317*	FGT		
Post	Ref.	1.0	Fat	3.8683 (0.2505,59.7455)	0.3327
CA153			Scattered Fibroglandular Tissue	Ref.	1.0
Negative	Ref.	1.0	Heterogeneous Fibroglandular Tissue	0.4836 (0.0878,2.6630)	0.4039
Positive	Inf. (0.0000,inf.)	0.9925	Extreme Fibroglandular Tissue	0.7191 (0.1308,3.9545)	0.7045

OR, odds ratio; CI, confidence interval; NA, not available. These variables were eliminated in the multivariable logistic regression model, so the OR and p-values were not available. *p < 0.05.

BMI, body mass index; TIC, time–signal intensity curve; CEA, Carcinoembryonic antigen; ADC, apparent diffusion coefficient; Ref, reference; Inf, infinite.

### Diagnostic Performance of Various MRI Parameters and Prediction Models in the Diagnosis of Breast Cancer and Benign Diseases

**Figure 1:**
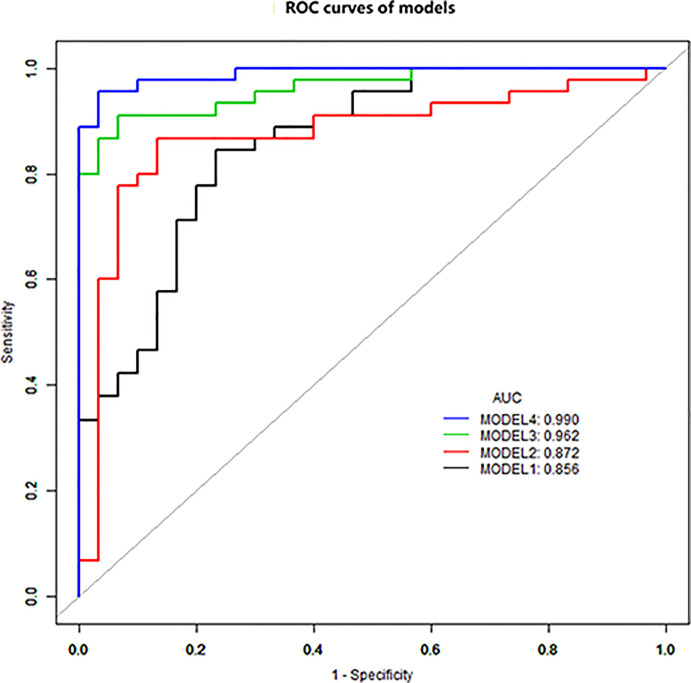
ROC curve analysis. Model 4 has the best diagnostic performance (AUC = 0.989), followed by models 3, 2, and 1 (AUC = 0.962, 0.872, and 0.856, all *p* < 0.05).

**Figure 2:**
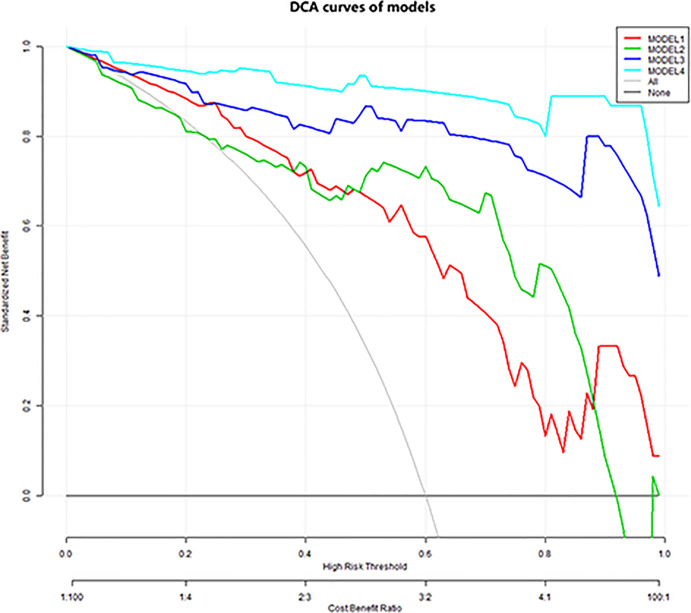
Decision curve analysis. The *y*-axis indicates net benefits to patients. The colorful lines represent the models, the gray line represents the hypothesis that all patients had breast cancer, and the black horizontal line represents the hypothesis that no patients had breast cancer. As showed in the curve, the net benefit of model 4 was better than that of the other models between threshold probabilities of 0% and 100%.

**Figure 3:**
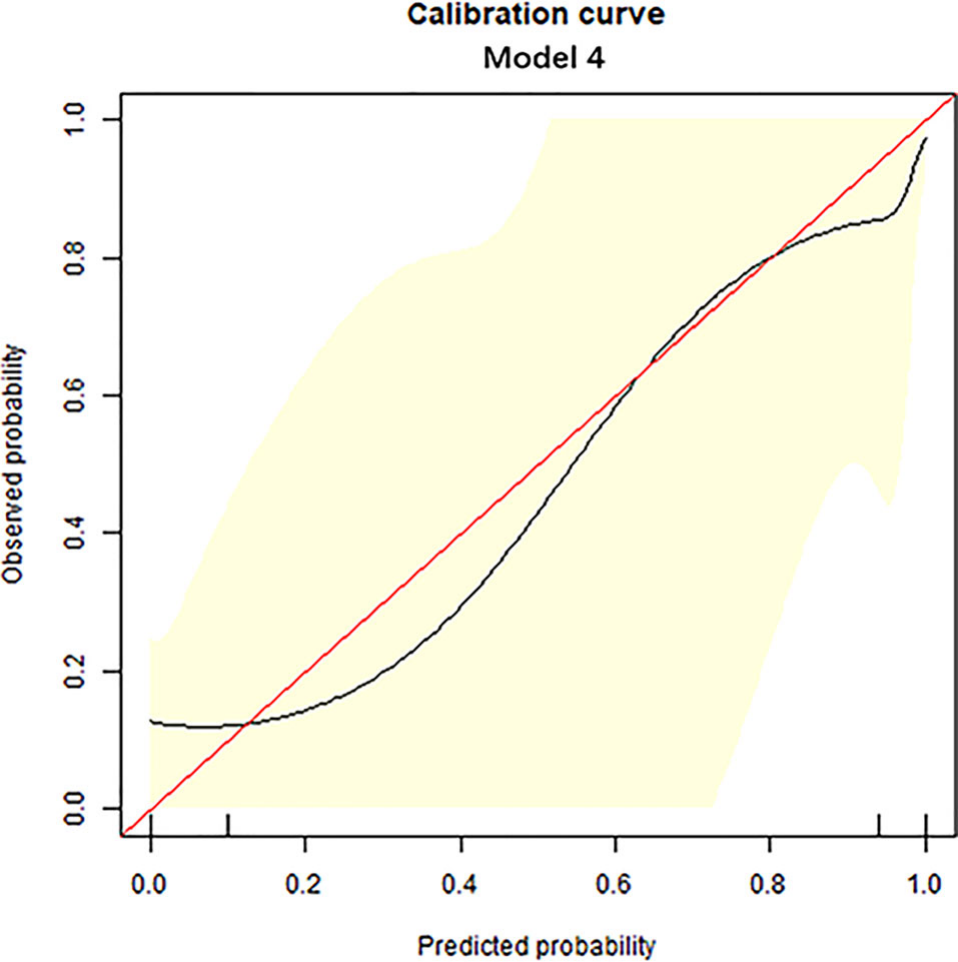
Calibration curve analysis. The calibration curve was used to show the relationship between the predicted value and the true value. The *x*-axis represents the predicted probability, while the *y-*axis represents observed probability. The red line represents the ideal calibration line, and the black line represents the predictive power of the nomogram. The closer the black line is to the red line, the better the predictive power of the model.

**Figure 4:**
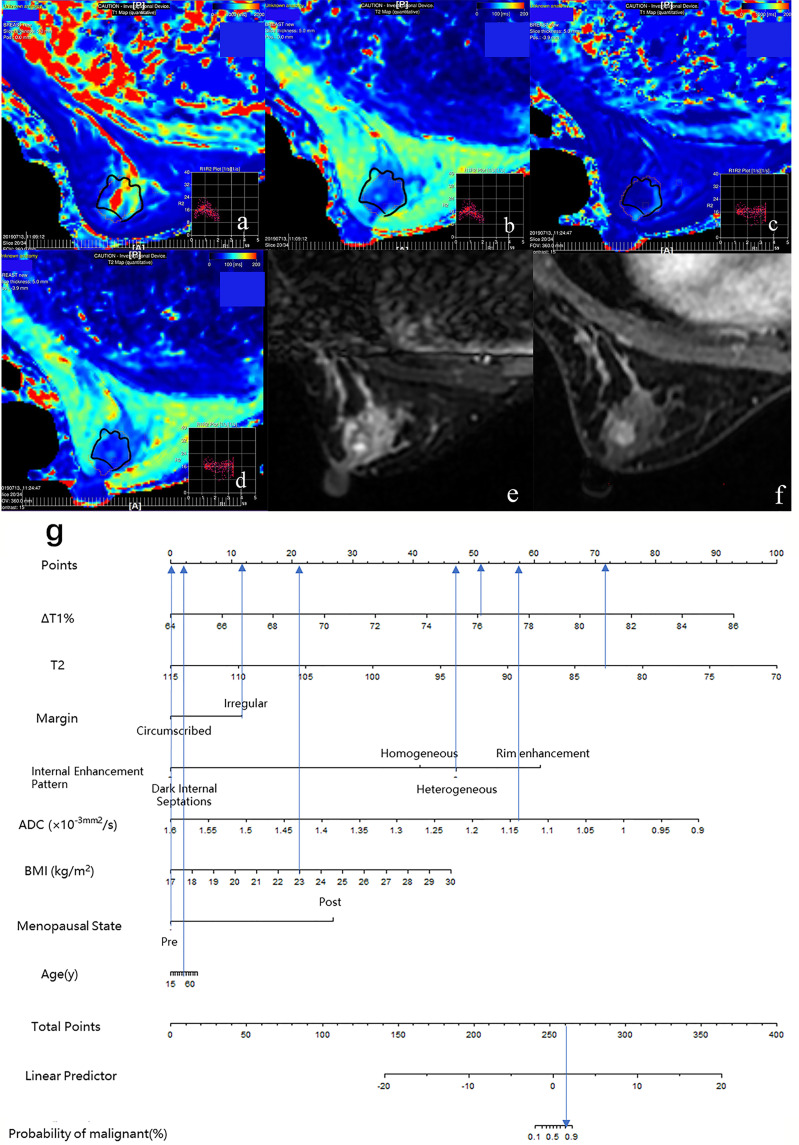
Case 1: Application of prediction model 4 with nomogram in distinguishing breast cancer and benign diseases in BI-RADS 4 lesions. Female, 46 years, premenopausal, BMI = 23, breast cancer, a–g. Image reconstruction with MAGiC, T1 map **(A)**, T2 map **(B)**, T1 enhancement map **(C)**, T2 enhancement map **(D)**, T2WI **(E)**, DCE **(F)**, Nomogram **(G)**. T1 = 1,349.77 ms, T1+ = 323.05 ms, ΔT1% = 76.07, T2 = 83.01 ms, T2+ = 78.03 ms, ΔT2% = 6.00%, ADC = 1.14 × 10^−3^ mm ^2^/s, irregular margin, and heterogeneous internal enhancement pattern. Total points = 261.80. Probability of malignant = 82.00%.

**Figure 5:**
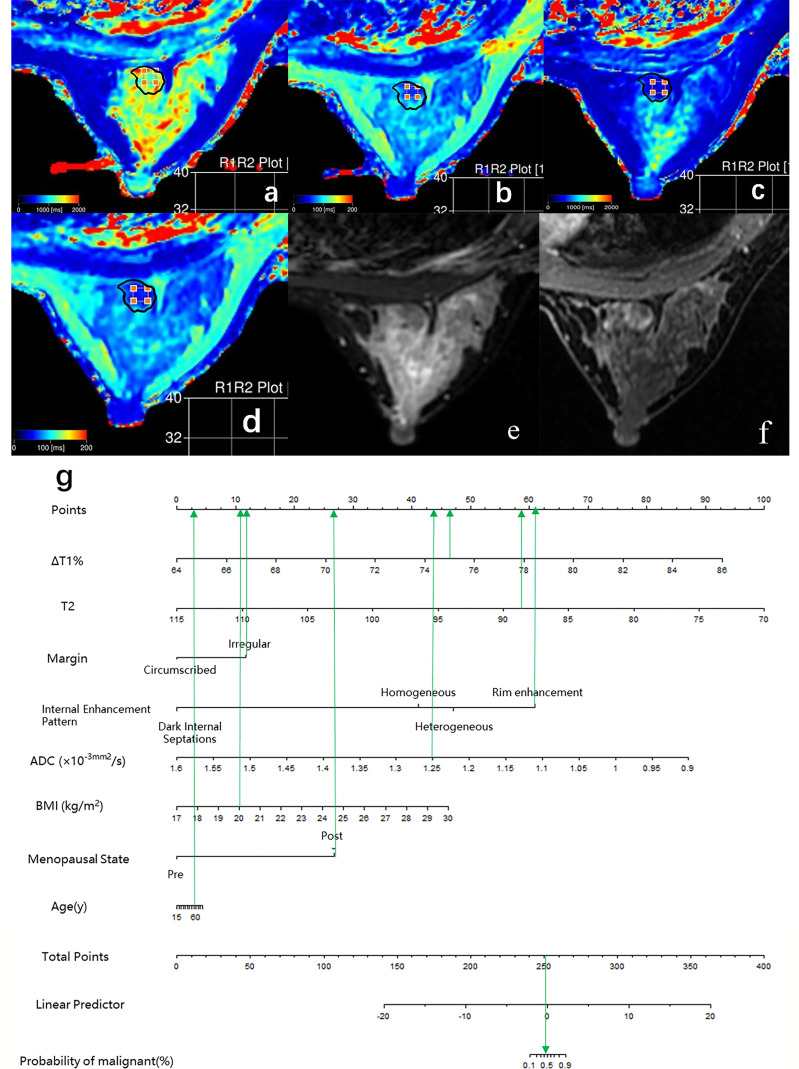
Case 2: Application of prediction model 4 with nomogram in distinguishing breast cancer and benign diseases in BI-RADS 4 lesions. Female, 50 years, postmenopausal, BMI = 20, fibroadenoma, a–g. Image reconstruction with MAGiC, T1 map **(A)**, T2 map **(B)**, T1 enhancement map **(C)**, T2 enhancement map **(D)**, T2WI **(E)**, DCE **(F)**, Nomogram **(G)**. T1 = 1,275.43 ms, T1+ = 315.03 ms, ΔT1% = 75.30, T2 = 88.75 ms, T2+ = 83.90 ms, ΔT2% = 5.46%, ADC = 1.25 × 10^−3^ mm ^2^/s, irregular margin and rim enhancement pattern. Total points = 253.18. Probability of malignant = 46.00%.

The diagnostic performance of each independent variable is listed in **Table 4**. Among the MRI quantitative parameters, T2 and ΔT1% were equivalent to ADC value (AUC = 0.798, 0.793, and 0.818; *p* = 0.809, 0.863). Four prediction models were established by combining the above independent parameters: model 1 (BI-RADS model, including all conventional MRI features recommended by BI-RADS lexicon), model 2 (relaxation time model, including ΔT1% and T2), model 3 (mpMRI model, including ΔT1%, T2, ADC, margin, and internal enhancement pattern), and model 4 (combined image and clinical model, including ΔT1%, T2, ADC, margin, internal enhancement pattern, age, BMI, and menopausal state). **Table 5** and **Figure 1** shows that model 4 has the best diagnostic performance (AUC = 0.989), followed by models 3, 2, and 1 (AUC = 0.962, 0.872, 0.856; all *p* < 0.05). Moreover, model 2 (relaxation model) showed higher diagnostic performance than ADC value alone (AUC = 0.872 *vs*. 0.818, *p* = 0.046), but similar to that of model 1 (AUC = 0.872 *vs*. 0.856, *p* = 0.814). DCA showed that the net benefit of model 4 was better than that of the other models between threshold probabilities of 0%–100% (**Figure 2**). The nomogram based on model 4 is shown in **Appendix 4**. The calibration curve of the nomogram provided evidence of optimal calibration (**Figure 3**, *P* = 1.21). The bootstrap resampling showed that the AUC was 0.979, and the sensitivity, specificity, positive predictive value (PPV), negative predictive value (NPV), and the accuracy of model 4 were 94.11%, 93.75%, 95.35%, 88.24%, and 92.21%, respectively. Typical cases are described in **Figures 4**, **5**.

**Table 4:** Diagnostic performance of different MRI parameters in the diagnosis of breast cancer and benign diseases.

Parameters	ROC							
	Best threshold	AUC	95% CI	Spe. (%)	Sen. (%)	PPV (%)	NPV (%)	Accuracy (%)
ΔT1%	75.470	0.793	0.684,0901	70.00	80.00	80.00	70.00	76.00
T2	87.120	0.798	0.685,0.911	86.67	77.78	89.74	72.22	81.33
ADC	1.195	0.818	0.718,0.917	83.33	77.78	87.50	71.43	80.00
Age	43.500	0.625	0.480,0.770	50.00	86.67	72.22	71.43	72.00
BMI	22.940	0.655	0.524,0.786	63.33	71.11	74.42	59.38	68.00
Margin			–	50.00	24.44	42.31	30.61	34.67
Internal Enhancement Pattern				33.33	93.33	67.74	76.92	69.33
Menopausal State				60.00	15.56	36.84	32.14	33.33

Sen, sensitivity; Spe., specificity; PPV, positive predictive value; NPV, negative predictive value.

**Table 5:** Diagnostic performance of different prediction models in the diagnosis of breast cancer and benign diseases.

Model		ROC						
	Variable	AUC	95% CI	Spe. (%)	Sen. (%)	PPV (%)	NPV (%)	Accuracy (%)
Model 1	BI-RADS	0.856	0.769,0.943	76.67	84.44	84.44	76.67	81.33
Model 2	ΔT1%, T2	0.872	0.782,0.962	86.67	86.67	90.70	81.25	86.67
Model 3	ΔT1%, T2, ADC margin, internal enhancement pattern	0.962	0.925,0.999	93.33	91.11	95.35	87.50	92.00
Model 4	ΔT1%, T2, ADC margin, internal enhancement pattern age, BMI, menopausal state	0.989	0.974,0.999	96.67	95.56	97.73	93.55	96.00

Sen., sensitivity; Spe., specificity; PPV, positive predictive value; NPV, negative predictive value.

Excellent intraobserver and interobserver agreement was noted for each parameter (intra/interclass coefficient (ICC)_intra_ and ICC_inter_ ≥ 0.995). The outcomes of the Bland–Altman plots and ICC were similar. All measurement points of the repeatability and reproducibility tests were within 2.5–97.5% limit of agreement (**Appendix 5**).

## Discussion

This study comprehensively analyzed the role of clinical and imaging features in identifying breast BI-RADS 4 lesions. Combining the variables from DCE-MRI, DWI, and syMRI, the mpMRI model (model 3) showed a good diagnostic performance for breast BI-RADS 4 lesions in this study. The sensitivity and specificity of diagnosis could be improved by adding clinical features. The combination model (model 4) could help the patients in avoiding unnecessary breast biopsies.

### Conventional MRI Characteristics in Differentiating Breast Cancer From Other Benign Diseases

The lesions that do not have typical malignant signs but sufficient suspicious manifestations are classified as BI-RADS 4. Kuhl et al. (25) pointed out that the following cases should be classified as BI-RADS 4. The shape (round or oval) and margin (circumscribed) of the lesion are not suspicious, but type III TIC curve (i.e., washout) is observed, or if the shape (irregular) and margin (irregular, spiculated) are suspicious, but kinetics enhancement is benign or borderline (the initial phase of TIC is slow to medium inflow, and the delayed phase is persistent enhancement or plateau, i.e., type I or II). Due to an overlap of the morphological and enhanced kinetic features, DCE-MRI cannot diagnose BI-RADS 4 lesions satisfactorily. In the current study, the AUC value of model 1, which combined the breast MRI descriptors recommended by BI-RADS lexicon, was only 0.856, and the sensitivity and specificity were 84.44% and 76.67%, respectively. The intraductal carcinoma *in situ*, low-grade invasive ductal carcinoma, and specific types of breast cancer accounted for about 57.78% of the lesions in this study. These low malignancy lesions did not show typical malignant signs, such as spiculated margin and fast heterogeneous contrast enhancement followed by a washout. On the contrary, fibroadenoma with rich blood supply or mucoid degeneration, intraductal papilloma, atypical ductal hyperplasia (ADH), and fibroadenomatous hyperplasia exhibited suspicious signs, such as irregular shape or margin, heterogeneous or rim enhancement pattern, and II or III TIC curve. These phenomena might lead to false positives and false negatives in the diagnosis.

Conventional MRI features have limitations in the diagnosis of BI-RADS 4 lesions. The current research showed that only irregular margins and the heterogeneous internal enhancement pattern on DCE-MRI were significantly associated with a breast cancer diagnosis. Lesions with irregular margins presented a 4.6-fold risk of malignancy [odds ratio (OR) = 1/0.217; 95% confidence interval (CI): 1.26–16.86] than regular lesions (*p* = 0.021). Lesions with heterogeneous internal enhancement pattern presented a 6.2-fold risk of malignancy (OR = 1/0.161; 95% CI: 1.01–38.17) than lesions with homogeneity (*p* = 0.049). We also observed that the malignant group showed more rim enhancement patterns than the benign group (31.11% *vs*. 20.00%). In addition, the dark internal septations only appeared in benign lesions, which were mainly characterized in the fibroadenoma with irregular margin, fast or medium enhancement in the initial phase of DCE, and plateau or washout in the delayed phase.

### Quantitative MRI Characteristics in Differentiating Breast Cancer From Other Benign Diseases

ΔT1%, T2, and ADC were discovered as independent variables for breast cancer in the multivariable logistic regression analysis. Thus, the degree of diffusion restriction of free water molecules and the ADC values are valuable in differentiating between benign and malignant lesions (6, 26–28). Due to continuous cell proliferation, the increased synthesis of macromolecular substances, such as proteins in the cytoplasm, and the release of a large number of necrotic substances, the extracellular space reduces, the content of bound water increases, and the diffusion of free water molecules is restricted. These factors decrease the ADC value of breast cancer. A meta-analysis of 14 articles showed that the cutoff range of ADC for differentiating between benign and malignant breast lesions was 0.92–1.61 × 10^−3^ mm ^2^/s (6), while another meta-analysis suggested a threshold of 1.23×10^−3^ mm ^2^/s (29). Also, the ADC value of the malignant lesion group was significantly lower than that of the benign lesion group (1.10 *vs*. 1.25 × 10^−3^ mm ^2^/s, *p* < 0.001). The best threshold of ADC is 1.195 × 10^−3^ mm (2)/s in our study, which is consistent with that of the previous study.

Furthermore, the difference in cell composition and microstructure also affects the magnetization characteristics of the tissue, which might be characterized by the relaxation time (T1 and T2). Some studies have shown that the difference in relaxation time was related to the content of free water (30–33). The higher the free water content, the longer the relaxation time. As described above, under the combined action of various factors, the ratio of free water and bound water is out of balance, resulting in decreased free water content and relaxation time (20, 21, 31). Previous studies have shown that relaxation times could distinguish between the breast neoplasms and other diseases or normal tissues, where the T2 relaxation time may be more distinctive than T1 (34). Reportedly, the T2 value of breast cancer is about 75.00–84.75 ms, which is lower than that of benign lesions (6, 21, 30, 35). Due to the difference in field strength, acquisition method, ROI, and pathological type, the T2 values obtained in each study were not identical but showed a similar trend. Liu et al. (20) showed that the T2 value of breast cancer was significantly lower than that of benign lesions (82.69 ms *vs*. 95.48 ms). Marina et al. (17) found that the T1/T2 rate of malignant lesions was significantly higher than that of benign lesions. The current study also showed that the T2 value of breast cancer was significantly lower than that of benign lesions (84.63 ms *vs*. 92.57 ms, *p* < 0.001), which was consistent with similar research results. With each unit (ms) increase in T2 value, the odds of the lesion being cancer decreased significantly (CI: 0.660–0.914; *p* = 0.002). Our results were consistent with those of Jung et al. as both studies used syMRI for imaging at 3.0 T, and a similar method was used for ROI delineation; therefore, the data were similar (84.34 ms *vs*. 84.75 ms).

Previous quantitative breast studies focused on the relaxation time before the injection of contrast agent (18, 20–23). One of the highlights of the present study is that we measured the T1 and T2 values pre- and post-contrast injection and calculated the relative change in T value between the two injections (ΔT%) to quantify the enhancement degree of the lesions. This method reduced the influence between different scanning devices, reflected the enhancement degree of the lesion, and provided more information than plain scanning. Especially for BI-RADS type 4 lesions, this method differentiated between benign and malignant lesions. Typically, breast cancer shows a rapid and stronger signal intensity increment after injection of contrast agents compared to most benign diseases (23, 36), which was confirmed by our study. ΔT1% in the malignant lesions group was significantly higher than that in the benign group (77.18% *vs*. 74.20%, *p* < 0.001). With each unit increase in ΔT1%, the odds of the lesion being cancer increased by 1.95-fold (OR = 1.947; 95% CI: 1.233–3.073; *p* = 0.004). The varied microcirculation structure of the tumor determines the difference in relaxation time before after contrast injection. A previous study has shown that breast cancer releases vascular endothelial growth factor (VEGF) and induces the formation of a large number of new capillary networks (37). These immature capillaries are disordered and tortuous with large diameter and high wall permeability, resulting in a large volume of contrast-carrying blood flow into the tumor. Therefore, breast cancer shows a shorter relaxation time value and a higher enhancement ratio.

Furthermore, we explored the role of relaxation time in the evaluation of BI-RADS 4 lesions. In terms of the contribution of a single variable to the diagnosis of breast cancer, T2 and ΔT1% were equivalent to ADC values (AUC = 0.798, 0.793, and 0.818; *p* > 0.05). The relaxation model (model 2) combined with T2 and ΔT1% showed a higher diagnostic performance than ADC value alone (AUC = 0.872 *vs*. 0.818, *p* = 0.046). The comparison between relaxation time and conventional MR features revealed that model 2 (relaxation time model) had a larger AUC than model 1 (0.872 *vs*. 0.856, *p* = 0.814) (BI-RADS model), albeit not significantly. However, when the acquisition time of syMRI and conventional MRI is similar—the total time of T1WI, T2WI, and DCE-T1WI is 10.3 min, the syMRI is 10.2 min—this finding is encouraging. SyMRI can obtain the synthetic contrast-weighted images of T1 and T2 pre- and post-contrast injection, which exhibit the shape and enhancement kinetic characteristics of lesions, and thus quantitative images for measuring a relaxation time. Thus, the qualitative and quantitative features could be obtained in 10 min using syMRI. Compared to the traditional breast MRI, this method simplifies the operation process of radiologists and provides additional quantitative relaxation information for differential diagnosis.

Since various imaging features can provide different aspects of information, the combination of multiple MRI features is called mpMRI, which has the potential to improve breast disease assessment and is being increasingly implemented in clinical settings (11, 14, 38). Several studies have proven that mpMRI combined with DCE-MRI, DWI, and MRS can further improve the specificity in the assessment of BI-RADS 4 lesions and avoid up to 36% of breast biopsies (29, 39–41). Our study showed that mpMRI (model 3) with DCE-MRI, DWI, and syMRI achieved a significantly higher diagnostic performance (0.962) compared to model 1 (0.856), model 2 (0.872), and ADC alone (0.818). These findings have gained increasing attention from the researchers interested in the role of MRI in the subdivision of BI-RADS 4 lesions. The mpMRI model has better diagnostic performance and provides objective quantitative parameters that facilitate the subdivision of BI-RADS 4 lesions.

### Clinical Characteristics in Differentiating Breast Cancer From Other Benign Diseases

Since the mpMRI model is useful, we considered adding clinical features to explore whether it could further improve the diagnostic performance. Several studies have shown that age, family history, obesity, and menopause are risk factors for breast cancer (42–44). The current study showed that age and BMI are significantly positively associated with the risk of breast cancer. With each unit (age, kg/m ^2^) increase in age and BMI, the odds of the lesion being cancer decreased significantly (95% CI: 1.007–1.119; 1.047–1.603; *p *= 0.026, 0.017). The risk of breast cancer in postmenopausal patients (OR = 1/0.217; 95 CI%: 1.26–16.86) was 3.95-fold higher than that in premenopausal patients (*p* = 0.032).

Previous studies have shown that endogenous steroid hormone (mainly estrogen) levels of sex steroids are associated with the risk of breast cancer and sustained tumor growth in women (45). Compared to the benign breast tissue, there is an increased uptake of estradiol by breast cancer cells from the circulation accompanied by increased androgen conversion in postmenopausal breast cancer tissue (46–49). In addition, a large amount of aromatase in adipose tissue further promotes the conversion of androgen. Both factors elevate the estrogen level in breast tissue, providing an optimal environment for the proliferation and growth of breast cancer cells and explaining the current results.

### Prediction Models in Differentiating Breast Cancer From Other Benign Diseases

With the addition of clinical features, the mp combination model (model 4) obtained the highest diagnostic performance (AUC = 0.989, all *p* < 0.05), and the sensitivity, specificity, PPV, NPV, and accuracy were improved to varying degrees. DCA was performed to evaluate the clinical utility of the models. As shown in **Figure 2**, the net benefit of model 4 was better than that of other models between a threshold probability of 0% and 100%. In order to increase the applicability in the clinical setting, this model was presented with a nomogram. Then, the malignancy probability of each lesion could be quantified by calculating the scores of each variable and adding these to obtain a total score. Thus, the contribution of each variable to breast cancer could be reflected intuitively and comprehensively. Finally, to verify the calibration of the nomogram, we applied a calibration curve. As shown in **Figure 3**, the true and the predicted curves are closely connected, indicating optimal predictability of the combined model in breast cancer.

## Limitations

Although the final results are gratifying, the present study has some limitations. First, the model established by multivariable logistic regression analysis is prone to overfitting, which could be addressed by setting up an independent model validation group but was limited by the sample size. Therefore, we adopted a bootstrap resampling method (1,000 times) for internal cross-verification and obtained satisfactory results. Nonetheless, the applicability of this model needs further verification. Second, although three-dimensional (3D) ROI contains a large amount of information, delineating two-dimensional (2D) ROI is efficient and clinically applicable considering the clinical work. In addition, similar studies have pointed out that for MAGiC images, 2D ROI from the single section of the lesion has better clinical efficacy than partial section, three sections, and whole lesion (50). Hence, we drew the 2D ROI along the margin of the tumor on a single section image with maximum tumor diameter. Third, to ensure the consistency and accuracy of the ROI delineation, only the lesions that appear as masses were included in this study. In future studies, we will explore the diagnostic value of relaxation time in non-mass lesions. Fourth, compared to the majority of invasive ductal carcinoma and fibroadenoma, our study is limited to a small number of ductal carcinoma *in situ* (DCIS), intraductal papilloma (IDP), and adenosis. This might limit the general applicability of the model in these subgroups.

## Conclusion

In conclusion, the mpMRI model with DCE-MRI, DWI, and syMRI is a robust tool for evaluating the malignant findings in BI-RADS 4 lesions. The addition of clinical features can further improve the diagnostic performance of this model.

## Data Availability Statement

The raw data supporting the conclusions of this article will be made available by the authors, without undue reservation.

## Ethics Statement

The study was approved by the Institutional Ethics Board of Yunnan Cancer Hospital (No. KY2019102). The patients/participants provided their written informed consent to participate in this study.

## Author Contributions

Conception and design: SS. Administrative support: YD and CL. Provision of study materials or patients: DZ and JZ. Collection and assembly of data: SS and LN. Data analysis and interpretation: SS, LN, and YL. Manuscript writing: All authors. Final approval of manuscript: All authors. All authors contributed to the article and approved the submitted version.

## Funding

This work was supported by (I) the Science and Technology Project of Yunnan Provincial Department of Science and Technology—Big Data Research on Breast Cancer Heterogeneity Using Magnetic Resonance Image Texture Analysis. NO.2018FE001(-066) and (II) the Scientific Research Project of Yunnan Provincial Department of Education—Multimodal functional imaging markers to predict the normalization of breast cancer tumor blood vessels. No. 2019J1280.

## Conflict of Interest

The authors declare that the research was conducted in the absence of any commercial or financial relationships that could be construed as a potential conflict of interest.

## Publisher’s Note

All claims expressed in this article are solely those of the authors and do not necessarily represent those of their affiliated organizations, or those of the publisher, the editors and the reviewers. Any product that may be evaluated in this article, or claim that may be made by its manufacturer, is not guaranteed or endorsed by the publisher.

## Supplementary Material

Supplementary Table 1 Histopathological Subtypes of the Patient Population.

Supplementary Table 2 Repeatability of relaxation time measurements by the same observer & Reproducibility of relaxation time measurements by two observers.

Supplementary Figure 1 The imaging protocol of MRI scanning.

Supplementary Figure 2 The flowchart of patient enrollment.

Supplementary Figure 3 Nomogram for prediction of breast cancer.
