# Supplementary material for: Multiparameter MRI Model With DCE-MRI, DWI, and Synthetic MRI Improves the Diagnostic Performance of BI-RADS 4 Lesions
Source: Front Oncol. 2021 Oct 15;11:699127. doi: 10.3389/fonc.2021.699127 (PMC8554332; doi:10.3389/fonc.2021.699127)
Supplement: Supplementary Table 1 — Histopathological Subtypes of the Patient Population. [file Table_1.docx]

| **Table 1.** Histopathological Subtypes of the Patient Population. | | | |
| --- | --- | --- | --- |
| Type | Malignant(n=45) | Type | Benign(n=30) |
| DCIS | 5 | Fibroadenomas | 13 |
| DCIS+IDC | 2 | Intraductal Papilloma | 4 |
| Invasive Ductal Carcinoma | 30 | Adenosis | 3 |
| I | 14 |  |  |
| II | 9 |  |  |
| III | 7 |  |  |
| Invasive Lobular Carcinoma | 2 | Benign phyllodes | 3 |
| Mucinous Adenocarcinoma | 3 | Atypical Ductal Hyperplasia (ADH) | 1 |
| Papillary carcinoma | 1 | Fibroadenomatous Hyperplasia | 2 |
| Metaplastic carcinoma | 1 | Aggressive Fibromatosis | 1 |
| Carcinoma with medulary features | 1 | Inflammatory | 3 |
| DCIS：ductal carcinoma in situ；IDC：invasive ductal carcinoma. | | | |
