# Supplementary material for: Multiparameter MRI Model With DCE-MRI, DWI, and Synthetic MRI Improves the Diagnostic Performance of BI-RADS 4 Lesions
Source: Front Oncol. 2021 Oct 15;11:699127. doi: 10.3389/fonc.2021.699127 (PMC8554332; doi:10.3389/fonc.2021.699127)
Supplement: Supplementary Table 2 — Repeatability of relaxation time measurements by the same observer & Reproducibility of relaxation time measurements by two observers. [file Table_2.docx]

After 2 weeks, two observers independently remeasured these data in the same way while blinded to their previous results. Two measurements were recorded as “A1 and A2”, “B1 and B2” respectively. The intraobserver agreement (i.e. repeatability) was assessed by two measurements made by the same observer. The interobserver agreement (i.e. reproducibility) was assessed by the average of two measurements (i.e. mA and mB) from two observers. The “mA” was used to represent the average of A1 and A2, and “mB” was the same as above.

|  | **Table 2.** Repeatability of relaxation time measurements by the same observer. | | | | | | |
| --- | --- | --- | --- | --- | --- | --- | --- |
|  | Repeatability | | Bland-Altman | |  | ICC | |
|  |  |  | M±SD  ms | LoA |  | ICC_intra_ | 95% CI |
|  | A1A2 | T1 | -54.24±13.69 | (-81.61,-26.86) |  | 0.999 | (0.999,0.999) |
|  |  | T1+ | -29.68±7.70 | (-45.22,-14.14) |  | 0.998 | (0.997,0.998) |
|  |  | T2 | -4.07±1.11 | (-6.29,-1.85) |  | 0.999 | (0.999,0.999) |
|  |  | T2+ | -4.17±1.13 | (-6.43,-1.91) |  | 0.999 | (0.998,0.999) |
|  |  | ADC | -0.03±0.02 | (-0.06,0.003) |  | 0.999 | (0.996,0998) |
|  | B1B2 | T1 | -21.14±11.98 | (-45.10,2.83) |  | 0.999 | (0.999,0.999) |
|  |  | T1 | -9.09±6.85 | (-22.80,-4.61) |  | 0.998 | (0.997,0.999) |
|  |  | T2 | -6.06±1.14 | (-8.34,-3.78) |  | 0.999 | (0.999,0.999) |
|  |  | T2 | -4.10±1.25 | (-6.59,-1.60) |  | 0.999 | (0.998,0.999) |
|  |  | ADC | -0.04±0.01 | (-0.03,0.006) |  | 0.999 | (0.997,0999) |
|  | ICC: Intraclass correlation coefficient; LoA: limits of agreement(2.5%~97.5%); CI: confidence interval; M±SD: Mean difference ± standard deviation; A1 A2: first and second measurements of observer A; B1 B2: first and second measurements of observer B | | | | | | |

| **Table 3.** Reproducibility of relaxation time measurements by two observers. | | | | | |
| --- | --- | --- | --- | --- | --- |
|  | Bland-Altman | |  | ICC | |
| Reproducibility  (mA mB) | M±SD  ms | LoA |  | ICC_inter_ | 95% CI |
| T1 | -302.56±24.93 | (-352.41,-252.71) |  | 0.998 | (0.998,0.999) |
| T1+ | -120.40±6.96 | (-134.32,-106.47) |  | 0.997 | (0.997,0.998) |
| T2 | -14.10±1.11 | (-16.32,-11.89) |  | 0.996 | (0.995,0.997) |
| T2+ | -14.95±1.64 | (-18.23,-11.67) |  | 0.997 | (0.996,0.998) |
| ADC | -0.12±0.05 | (-0.10,0.02) |  | 0.997 | (0.997,0.998) |
| ICC: Intraclass correlation coefficient; LoA: limits of agreement(2.5%-97.5%); CI: confidence interval; M±SD: Mean difference ± standard deviation; mA: the average of two measurements from observers A; mB: the average of two measurements from observers B | | | | | |
