# Supplementary figures and images for: Multiparameter MRI Model With DCE-MRI, DWI, and Synthetic MRI Improves the Diagnostic Performance of BI-RADS 4 Lesions
Source: Front Oncol. 2021 Oct 15;11:699127. doi: 10.3389/fonc.2021.699127 (PMC8554332; doi:10.3389/fonc.2021.699127)

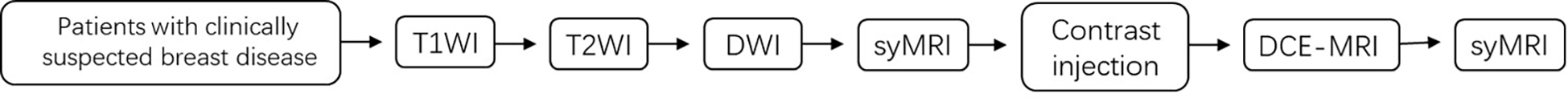

Supplement: Supplementary Figure 1 — The imaging protocol of MRI scanning. [file Image_1.tif]

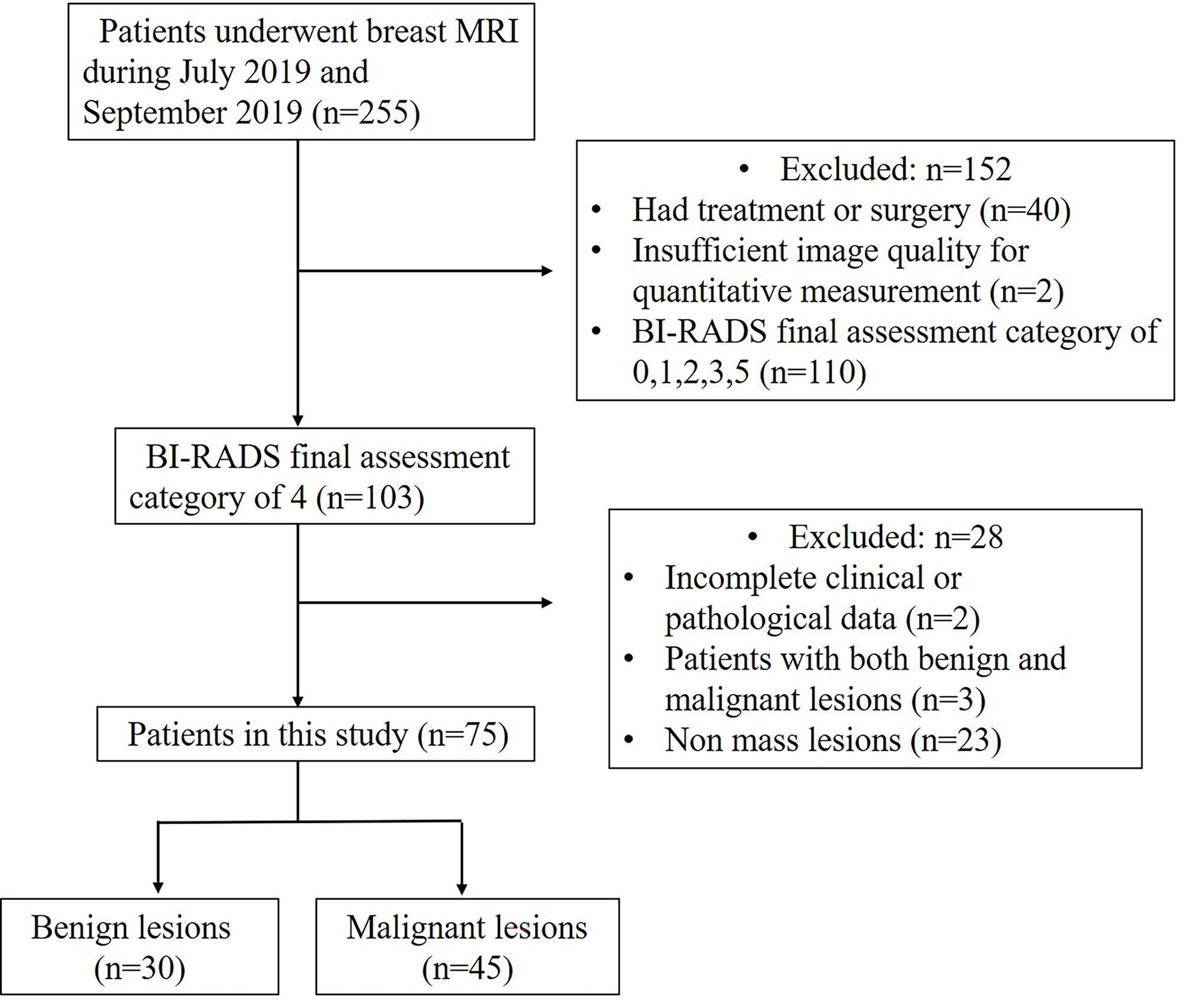

Supplement: Supplementary Figure 2 — The flowchart of patient enrollment. [file Image_2.tif]

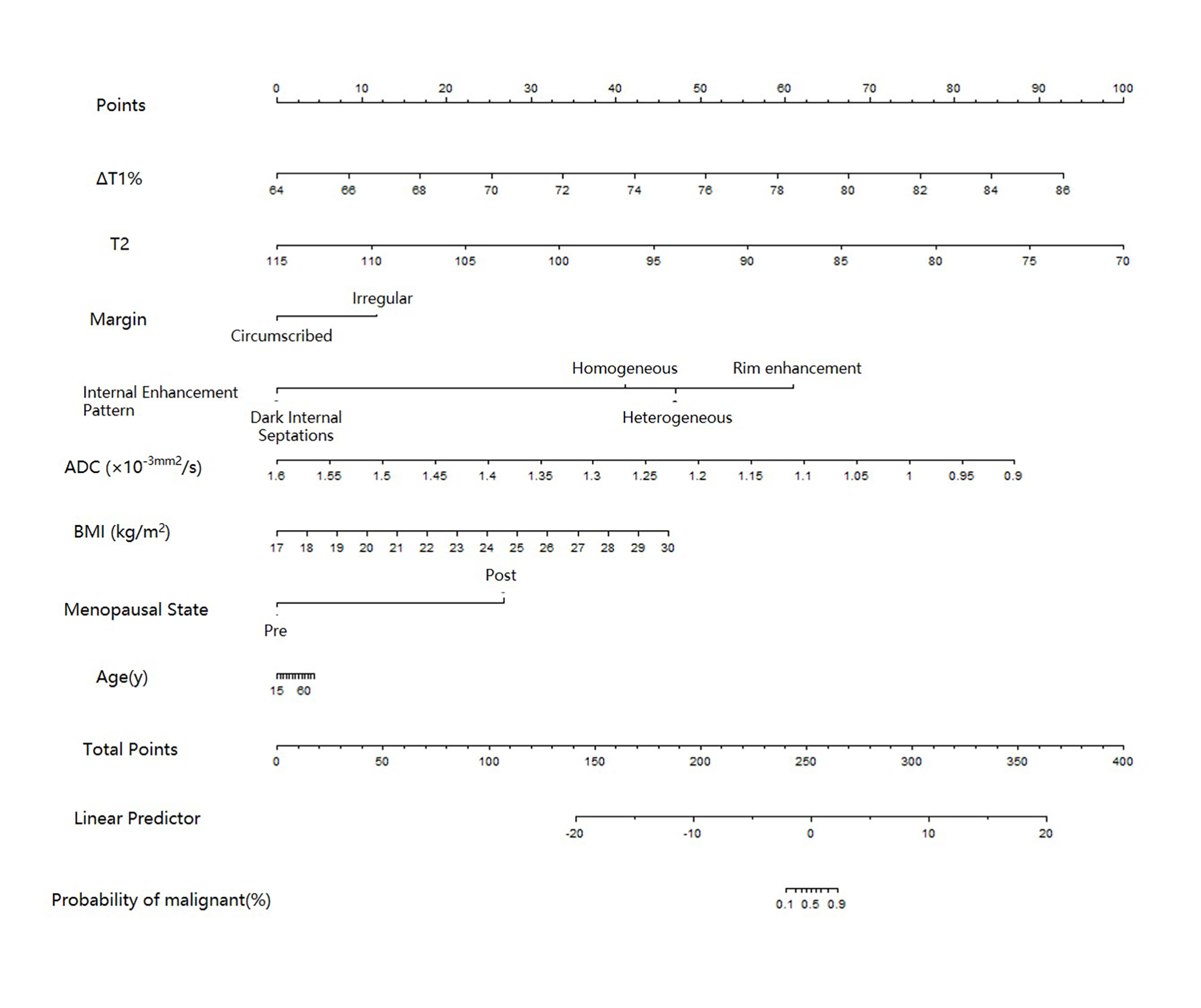

Supplement: Supplementary Figure 3 — Nomogram for prediction of breast cancer. [file Image_3.tif]
